# How to do a 3D uterus ultrasound?

**DOI:** 10.1007/s00404-023-06923-y

**Published:** 2023-02-17

**Authors:** Gertruda Jonaityte, Karl Oliver Kagan, Natalia Carmen Prodan, Markus Hoopmann

**Affiliations:** Department of Obstetrics and Gynaecology, University of Tuebingen, Calwerstrasse 7, 72076 Tübingen, Germany

**Keywords:** Three-dimensional (3D) ultrasound, Uterine anomalies, Gynecological ultrasound

## Abstract

Three-dimensional (3D) ultrasound is an invaluable tool in the detection and evaluation of many uterine anomalies and improves upon the traditional approach of two-dimensional (2D) ultrasonography. We aim to describe an easy way of assessing the uterine coronal plane using the basic three-dimensional ultrasound in everyday gynecological practice.

## What does this study add to the clinical work

**Table:** 

This study shows an easy way for using three-dimensional (3D) ultrasound in the detection and evaluation of many uterine anomalies in everyday clinical work.	

A coronal plane view, which encompasses the entire endometrial cavity is usually impossible to obtain using a traditional 2D ultrasound. However, it can be easily displayed using 3D ultrasound reconstruction. Many uterine anomalies are initially suspected on 2D ultrasound. 3D ultrasound reconstruction can then be used to define the lesion much more accurately and is recognized as the standard method for the diagnosis of congenital uterine malformations. Several publications have demonstrated the high level of accuracy in diagnosing and defining uterine anomalies using such a standardized approach. In the hands of an experienced sonographer, the diagnosis of uterine anomalies by 3D ultrasound has been shown be as accurate as an MRI [1]. Additionally, 3D ultrasound performs better than routine 2D ultrasound in diagnosing and defining endometrial pathologies, leiomyomata uteri, and is better in localization of an IUD [2, 3]. The Ultrasound Societies of German speaking countries have based their latest guidelines and quality requirements with these realities in mind [4, 5]. An additional benefit of having a stored 3D volume available is that it can be reviewed offline by other experts to provide a second opinion. This increases the diagnostic value of the examination [6].

The goal of the following presentation is to summarize the best method for obtaining a uterine volume for diagnostic purposes in an everyday gynecological practice. The focus is on reconstruction of the coronal view of the entire endometrial cavity as this allows for the best assessment of the presence and the type of a congenital uterine malformation. Optimally, the endometrial thickness should be at least 5 mm, or the timing of the examination should fall between day 17–25 of the menstrual cycle [6, 7]. Underlying conditions such as leiomyomata or adenomyosis and the presence of an IUD may compromise the evaluation of the endometrial contour. Examination of the endometrial cavity during pregnancy or menstrual bleeding should be avoided for the same reason (Tables 1, 2, 3, 4 and 5).

**Fig. 1:**
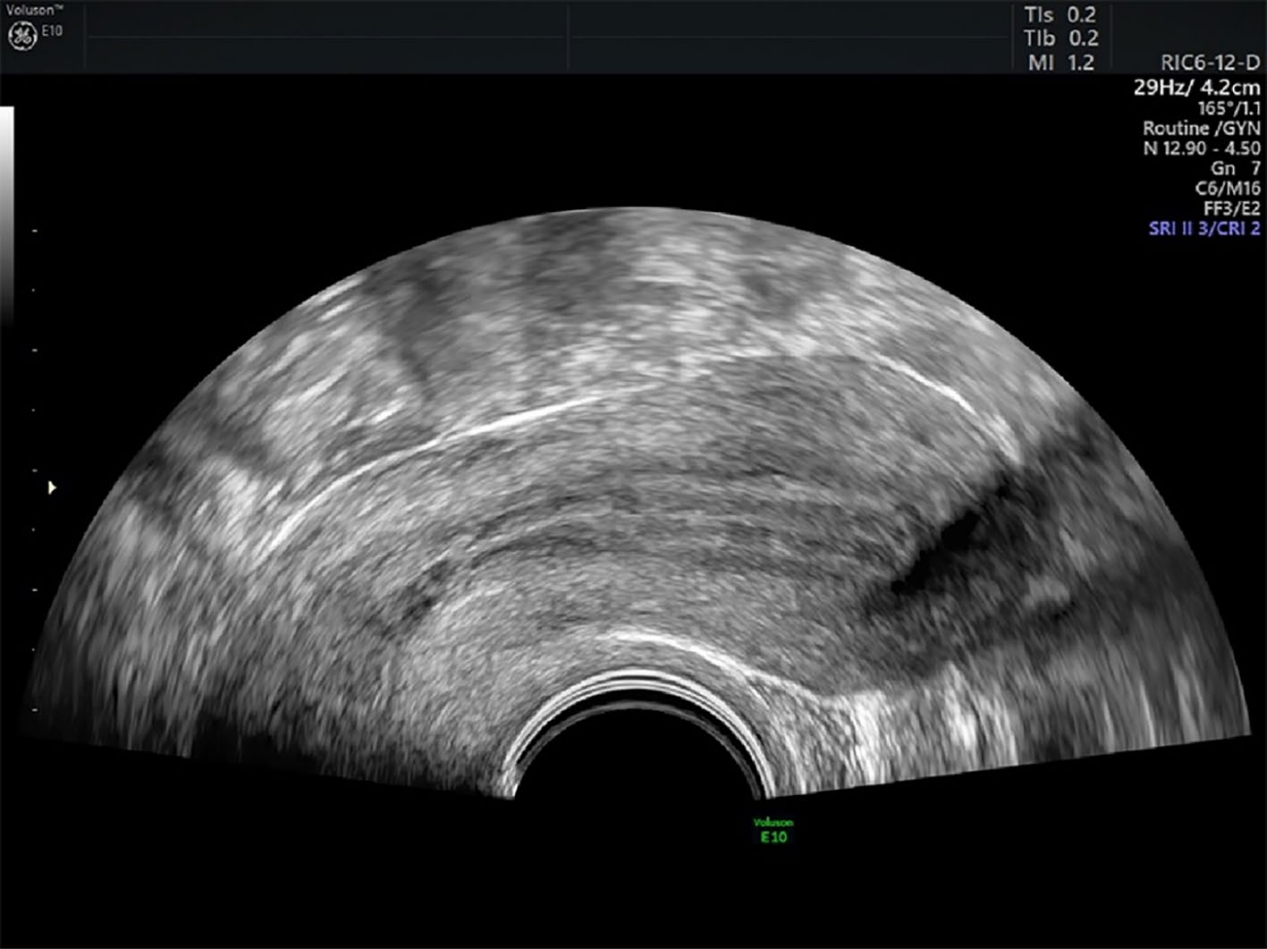
Midsagittal plane of the uterus

Step 1: Obtaining uterus midsagittal plane in 2D (Table 1; Figure 1)

**Table 1:** Requirements for optimal plane

Requirements for optimal plane
Sufficient magnification
Continuity of endometrium in midsagittal plane

**Fig. 2:**
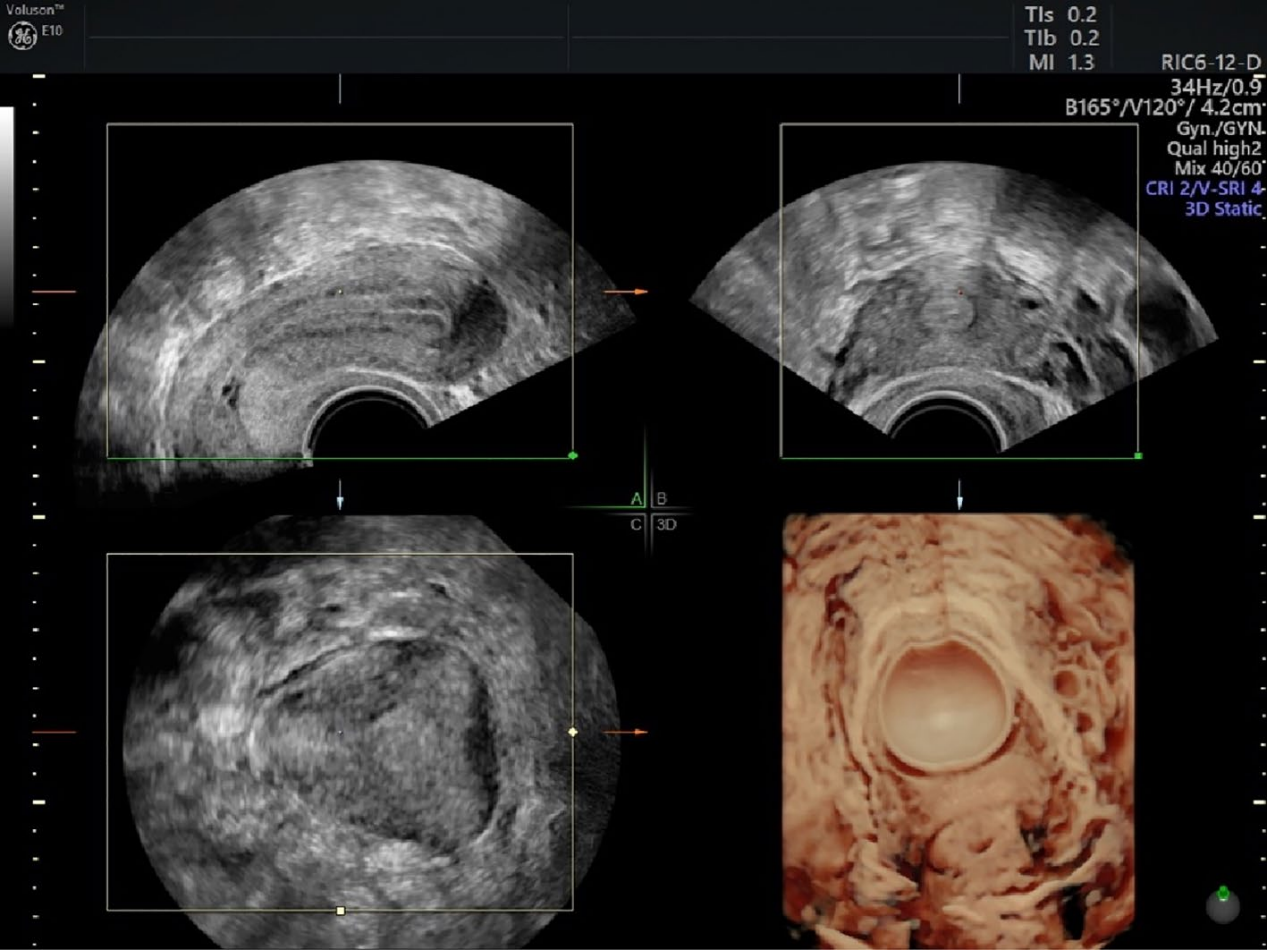
Multi-planar view contains four separate planes: midsagittal (**A**), transversal (**B**), coronal (**C**) and 3D view of the uterus

Step 2: Acquisition of standardized multi-planar view. (Table 2, Figure 2)

**Table 2:** Suggestions for an optimal acquisition

Suggestions for an optimal acquisition
Patient should hold breath and do not move during the process
Maximum sweep angle of 180°

**Fig. 3:**
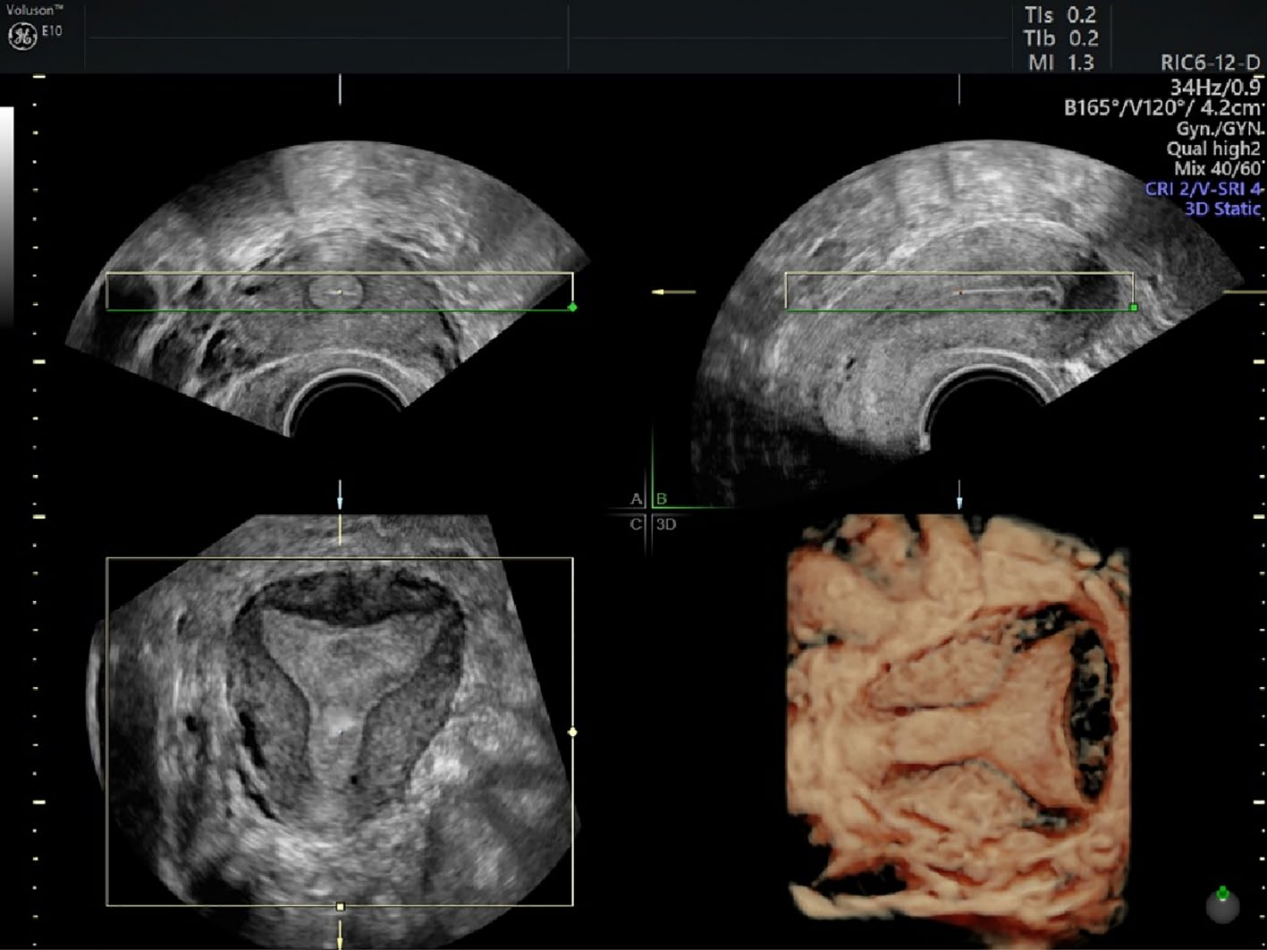
Multi-planar view of the uterus

Step 3: Adjusting the multi-planar view using the “Z rotation” technique [8]. (Table 3, Figure 3)

**Table 3:** Steps for the Application of the Z Technique

Steps for the Application of the Z Technique
Step 1. Place the reference/rotational point in the midlevel of the endometrial stripe in the sagittal plane
Step 2. Use the Z rotation to align the long axis of the endometrial stripe along the horizontal axis in the sagittal plane of the uterus
Step 3. Place the reference/rotational point in the midlevel of the endometrial stripe in the transverse plane
Step 4. Use the Z rotation to align the endometrial stripe with the horizontal axis in the transverse plane of the uterus
Step 5. After step 4, the midcoronal plane of the uterus will be displayed in coronal plane, apply Z rotation on coronal plane to display the midcoronal plane in the traditional orientation

**Fig. 4:**
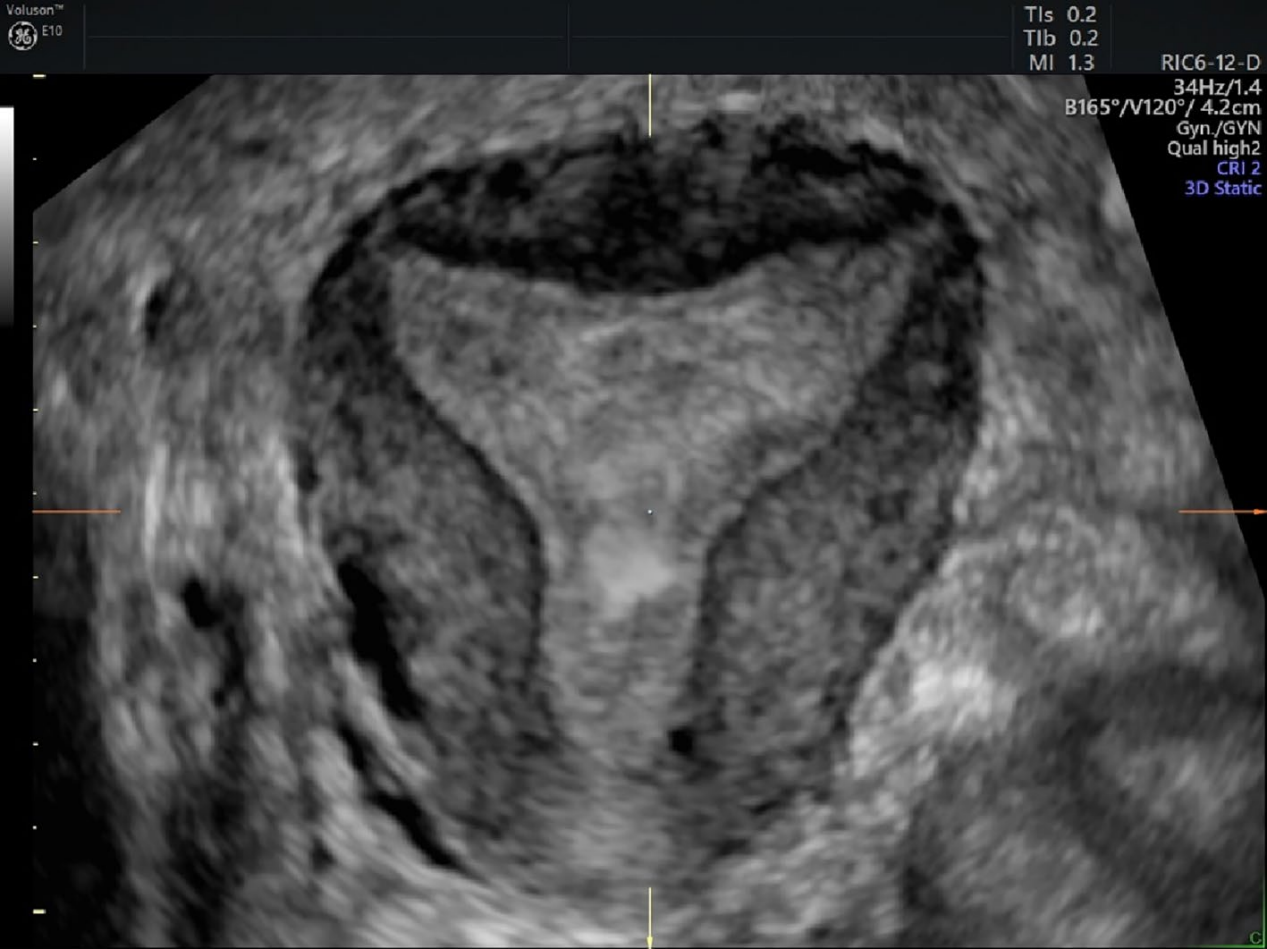
Coronal view of the uterus

**Fig. 5:**
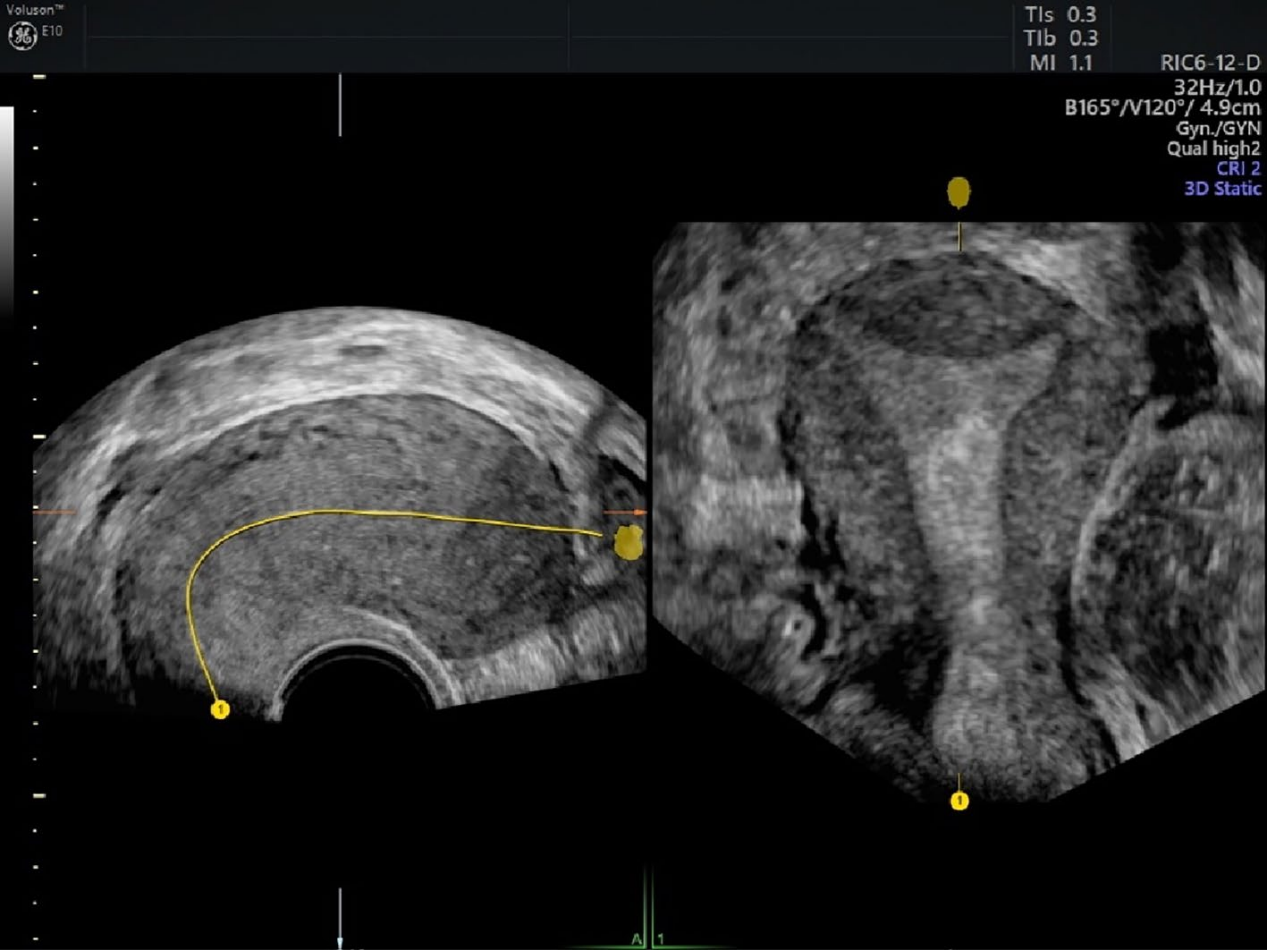
Coronal view of the uterus using trace line in “omni view” function

**Fig. 6:**
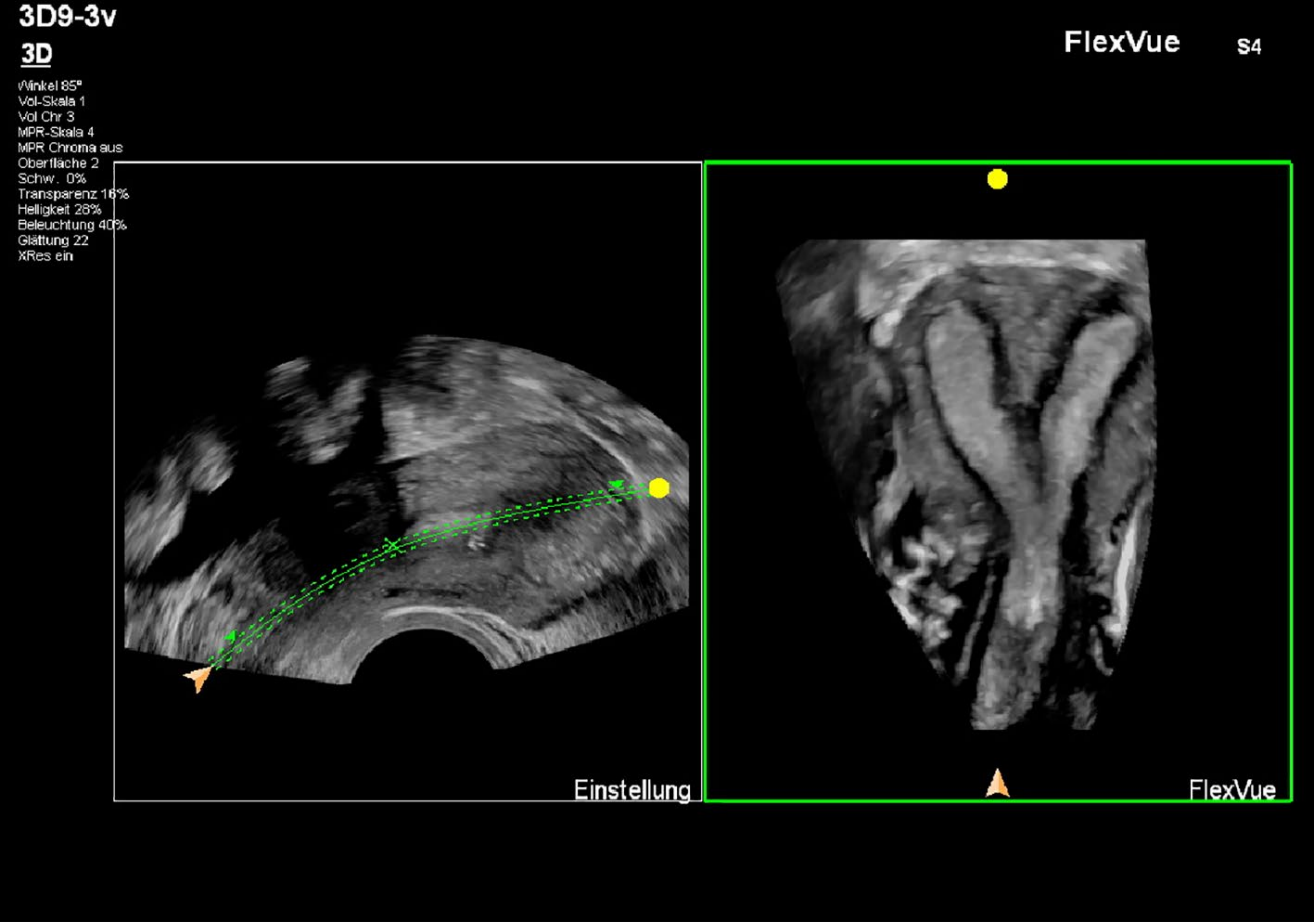
Coronal view of a septate uterus using trace line in “omni view” function

Step 4: Obtaining correct coronal plane of the uterus. (Table 4, Figures 4, 5, 6)

**Table 4:** Suggestions for an optimal coronal plane

Suggestions for an optimal coronal plane
Visualization of endometrial-myometrial junction zone
If possible, visualization of tubal insertion

In case of extreme retroflexion or other untypical positions of the uterus you can also use the “omni view” function with a trace line. This is a means to optimize the view of uterine cavity including the cervix.

Using these steps, you will easily obtain the basic three-dimensional ultrasound of the uterus coronal plane.

**Fig. 7:**
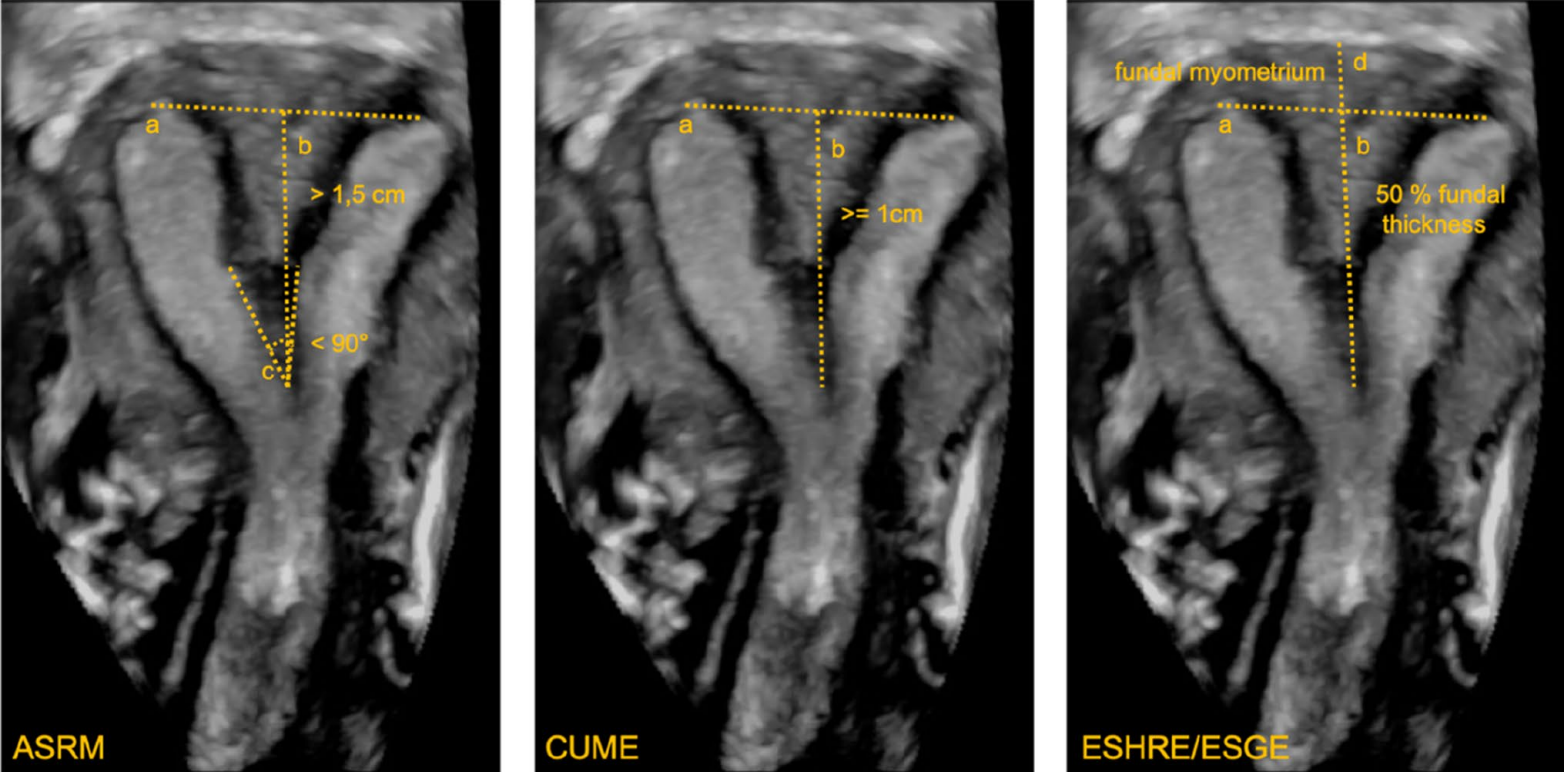
Septate uterus according to the three different societies ASRM american society for reproductive medicine, CUME congenital uterine malformation by experts, ESHRE/ESGE European society of human reproduction and embryology/European society for gynecological endoscopy **a** intercornual line **b** internal fundal indentation** c** indentation angle** d** uterine-wall thickness

Currently, there is no clear consensus among three leading societies (ASRM [9], ESHRE/ESGE [10], CUME [11]) on the classification of congenital uterine anomalies, in particular, of a septate vs. normal uterus. Due to the lack of a consensus it is advised to choose one of the classifications and apply it in everyday gynaecological practice. Following table (Table 5 and Figure 7) shows overview of the societies on the definition of a septate uterus.

**Table 5:** Classification of congenital uterine anomalies among three leading societies (ASRM [9], ESHRE/ESGE [10], CUME [11])

Society	Criteria for septate uterus
ASRM-2016 [9]	Internal fundal indentation depth ≥ 1.5 cm Indentation angle < 90° External fundal indentation depth < 1 cm
ESHRE/ESGE-2016 [10]	Internal fundal/uterine indentation depth > 50% of uterine-wall thickness External fundal indentation depth < 50% of uterine-wall thickness When uterine-wall thickness measured above interostial/intercornual line
CUME-2018 [11]	Internal fundal indentation depth ≥ 1 cm Indentation angle < 140° External fundal indentation depth < 1 cm

## Data Availability

Data sharing not applicable to this article as no datasets were generated or analysed during the current study.
